# Viscoelastic Characterization of Parasagittal Bridging Veins and Implications for Traumatic Brain Injury: A Pilot Study

**DOI:** 10.3390/bioengineering8100145

**Published:** 2021-10-18

**Authors:** Silvia García-Vilana, David Sánchez-Molina, Jordi Llumà, Ignasi Galtés, Juan Velázquez-Ameijide, M. Carmen Rebollo-Soria, Carlos Arregui-Dalmases

**Affiliations:** 1 Escola d’Enginyeria de Barcelona Est, Universitat Politècnica de Catalunya, Av. Eduard Maristany, 16, 08019 Barcelona, Spain; silvia.garcia.vilana@upc.edu (S.G.-V.); jordi.lluma@upc.edu (J.L.); juan.velazquez@upc.edu (J.V.-A.); 2 Institut de Medicina Legal i Ciències Forenses de Catalunya, G.V. Corts Catalanes, 111, 08014 Barcelona, Spain; ignasigaltes@gmail.com; 3 Departament de Psiquiatria i de Medicina Legal, Universitat Autònoma de Barcelona, Cerdanyola del Vallès, 08193 Barcelona, Spain; 4 Instituto de Medicina Legal de Aragón, Irene Izárbez, 22005 Huesca, Spain; mcrebollos@aragon.es; 5 Centro Zaragoza, Crtra. 232, km.273, 50690 Pedrola, Spain; carlos.arregui@centro-zaragoza.com

**Keywords:** bridging veins, TBI, tissue characterization, biomechanics, strain rate dependent materials, viscoelasticity

## Abstract

Many previous studies on the mechanical properties of Parasagittal Bridging Veins (PSBVs) found that strain rate had a significant effect on some mechanical properties, but did not extensively study the viscoelastic effects, which are difficult to detect with uniaxial simple tensile tests. In this study, relaxation tests and tests under cyclic loading were performed, and it was found that PSBVs do indeed exhibit clear viscoelastic effects. In addition, a complete viscoelastic model for the PSBVs is proposed and data from relaxation, cyclic load and load-unload tests for triangular loads are used to find reference values that characterize the viscoelastic behavior of the PSBVs. Although such models have been proposed for other types of blood vessels, this is the first study that clearly demonstrates the existence of viscoelastic effects from an experimental point of view and also proposes a specific model to explain the data obtained. Finally, this study provides reference values for the usual viscoelastic properties, which would allow more accurate numerical simulation of PSBVs by means of computational models.

## 1. Introduction

The importance of the TBI at the global level cannot be overlooked. Every year, about 70 million people worldwide are afflicted with some form of TBI, with the global incidence reaching about 940 cases per 100,000 people [1]. Among the factors leading to different types of TBI, a particularly pressing one is SDH produced by the rupture of some types of PSBVs [2,3]. In particular, the superior sagittal sinus-bridging veins, which link the central sagittal sinus to the upper part of the brain mass, have been identified as a highly critical region prone to producing SDHs of some severity [4,5,6,7].

For this reason, detailed and accurate studies of the mechanical properties of the collagenous tissue constituting the PSBVs are important, and should be updated using better and more accurate measurement techniques. For example, one influential paper detected a negative correlation of ultimate stress and strain rate [4]; however, a later paper showed a positive correlation [7], although neither author found the correlation to be statistically significant. The issue was recently closed when the use of more accurate digital measurement systems found a positive and significant correlation [3]. Although strain-rate dependent behavior for some mechanical properties have been studied by several authors [4,6,7,8], there are still few studies in the literature properly investigating the viscoelastic behavior of PSBVs, including the measurement of relaxation times or preconditioning under cyclic loading [3,9]. An important advance is found in [10] which examines preconditioning by cyclic loading and fatigue. However, although some work has been done for other types of blood vessels [11], no specific and complete viscoelastic model has yet been proposed for the PSBVs. The aim of this study is to contribute to filling the gap in viscoelastic characterization, using a more complex series of tests including load-unload cycles, stress relaxation cycles and fast repeated loading cycles, following the line of other works on the characterization of viscoelastic behavior of biological tissues [12,13,14].

This work shows that, the viscoelastic behavior of the PSBVs is clearly observable, measurable and quantifiable when observing the rehology of the tissue in a series of more complex tests, such as cyclic-load and relaxation tests, instead of conventional simple uniaxial tests, where the contribution of the viscoelastic effect to the mechanical behavior is difficult to quantify [3]. Such viscoelastic behavior can be used to improve both the understanding of injury mechanisms [15,16], and the computational models used to assess the probability of severe injuries.

## 2. Data and Methods

### 2.1. Material and Specimen Preparation

For this study, different sections of the meningeal-cortex space were obtained from four autopsies of post-mortem human subjects (PMHS) conducted at the Forensic Pathology Service of the Legal Medicine and Forensic Science Institute of Catalonia (FPS/IMLCFC). The study was approved by the Research Committee of the IMLCFC. None of the subjects had been previously diagnosed with any blood vessel pathology.

Once received, the meningeal sections were kept refrigerated for, at most, 96 h in airtight containers that maintained the natural degree of hydration. The sections of the meningeal-cortex space included the meninges, the subarachnoid space, and the upper part of the cerebral cortex. From those four sections, six PSBVs were carefully dissected (more than one PSBV was dissected from two sections). After dissection, the PSBVs were kept in contact with towel tissue soaked in phosphate-buffered saline solution. Twelve to twenty-four hours before testing, the PSBVs were placed in a refrigerator at 2 ${}^{\circ }$C and before testing they were allowed to come to room temperature for one hour.

### 2.2. Cyclic and Relaxation Tests

The tests were performed with an Allround-Table-Top Universal Test Machine (UTM) Zwick-Roell^®^ and load was measured with a 20N load cell HBM^®^. Special fixtures were used for the PSBVs tests and the displacement was carefully measured with a digital control unit attached to the UTM (due to the small dimensions of the cross-section of the PSBVs, no significant local effects appear in the displacement measures). The specimens were kept in physiological saline solution at 5 ${}^{\circ }$C to minimize tissue degradation during the testing.

**Figure 1:**
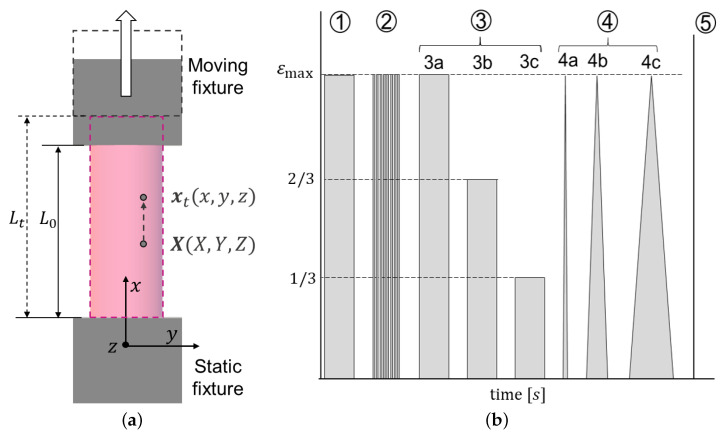
(**a**) Experimental setting measurements: the coordinate system is centered on the static fixture with the *x* axis parallel to the load direction, and a position change from the initial X to the deformed $x_{t}$ configuration is represented. (**b**) Cyclic and relaxation test stages scheme: (1) load-holding to maximum strain ($\varepsilon_{\max}$), (2) fast load/unload cycle stage to maximum strain, (3) load-holding to $\varepsilon_{\max}$, 2/3$\cdot \varepsilon_{\max}$ and 1/3$\cdot \varepsilon_{\max}$, (4) load and unload to $\varepsilon_{\max}$ at 0.01,0.1,1 s${}^{-1}$ and (5) load to fracture.

During the tests, a digitally displacement-controlled maximum strain $\varepsilon_{\max}$ was applied and the force experienced by each PSBV specimen was measured very accurately. In order not to cause permanent damage to the specimens, $\varepsilon_{\max}$ was selected as 20% of the average ultimate strain, determined in previous tensile studies of PSBVs [3]. The sequence of stages of the loading process was similar to that used in other soft tissue studies such as [12]. The different stages are as follows: (1) load-hold at $\varepsilon_{\max}$, (2) rapid load-unload cycles at $\varepsilon_{\max}$, (3) three stages of load-hold at $\varepsilon_{\max}$, 2/3$\cdot \varepsilon_{\max}$ and 1/3$\cdot \varepsilon_{\max}$, (4) three load-unload stages at $\varepsilon_{\max}$ at stretch rates of 0.01, 0.1 and 1 s${}^{-1}$ and (5) load to failure. Figure 1b shows a schematic of the above stages. The tests were performed at a speed of 50 mm/s (except for stage 4) and the holding time and the time between load stages were 50 s, allowing the specimen to recover completely.

In this study, each PSBV is modeled as a homogeneous, transversely isotropic, hollow cylinder with constant cross-section [3,4,5,7]. The coordinate axes have their origin upon the static bottom fixture; the *X* axis is aligned with the (longitudinal) force, while the *Y* and *Z* axes are contained in the PSBV cross-section. Thus, the stretch for each time instant is easily determined as $\lambda_{t}=\ell_{t}/\ell_{0}=1+\delta_{t}/\ell_{0}$ where $\ell_{0}$ is the initial length, $\ell_{t}$ the instantaneous length and the displacement $\delta_{t}=\ell_{t}-\ell_{0}$ has been digitally measured. Let $X_{t}=\left(X_{t},Y_{t},Z_{t}\right)$ be the coordinates in the initial configuration and $x_{t}=\left(x_{t},y_{t},z_{t}\right)$ in the deformed configuration, the deformation of the cylinder is expressed as

(1) $$x_{t}=X\lambda_{t},\;y_{t}=Y{\left[1-\bar{\nu }\left(\lambda_{t}^{2}-1\right)\right]}^{1/2},\;z_{t}=Z{\left[1-\bar{\nu }\left(\lambda_{t}^{2}-1\right)\right]}^{1/2}$$

where $\bar{\nu }\left(\lambda_{t}\right)$ represents the Poisson effect. Thus, being $F_{t}=\partial x_{t}/\partial X$ the deformation gradient tensor, the (Green-Lagrangian) strain tensor $\varepsilon =(F_{t}^{T}F_{t}-1)/2$ is obtained as

(2) $$\varepsilon \left(t\right)=\frac{1}{2}\left[\begin{array}{ccc} \lambda_{t}^{2}-1 & 0 & 0\\ 0 & -\bar{\nu }\left(\lambda_{t}\right)\left(\lambda_{t}^{2}-1\right) & 0\\ 0 & 0 & -\bar{\nu }\left(\lambda_{t}\right)\left(\lambda_{t}^{2}-1\right) \end{array}\right]$$

On the other hand, the force $F_{t}$ has been measured for each time instant during the whole test. From the force, the (second Piola-Kirchhoff) stress tensor σ is obtained:

(3) $$\sigma \left(t\right)=\left[\begin{array}{ccc} \frac{F_{t}}{\lambda_{t}A_{0}} & 0 & 0\\ 0 & 0 & 0\\ 0 & 0 & 0 \end{array}\right]$$

being $A_{0}$ the initial (undeformed) cross-section of the PSBV.

### 2.3. Quasi-Linear Viscoelastic Model

Many previous studies had used uniaxial tensile tests on PSBVs, showing that an elastic model reasonably explains the results obtained in this type of test. For this reason, the collagenous tissue of PSBVs has been treated simply as an elastic material. However, the cycle tests and the relaxation tests performed show that there is a clear viscoelastic effect on the mechanical behavior of PSBVs and that, therefore, viscoelastic models would work better for more general situations than simple traction.

In this study, a viscoelastic model of type QLVE [13,17] will be used to model the data. In the QLVE model, the mechanical response is divided into a strain-rate dependent part and a strain-rate independent part: $\sigma_{x}=\sigma_{x}^{\left(e\right)}+\sigma_{x}^{\left(v\right)}$, using a separable relaxation function $R\left(\varepsilon_{x},t\right)=G\left(t\right)\cdot \partial \sigma_{x}^{\left(e\right)}/\partial \varepsilon_{x}$[13,18], where G(t) is the relaxation function, given by the well-known Prony series [19,20,21,22]:

(4) $$G\left(t\right)=1+\sum_{k=1}^{N}g_{k}e^{-t/\tau_{k}}$$

with $g_{k}$ being the “weights” of each *k*-term and $\tau_{k}$ being the relaxation times, both to be obtained from experimental trials. The value of *N* depends on the needs of the setting, in Section 3.1 it is shown that N=3 is sufficient to represent the data in this study. With these assumptions, the axial strain (using the second Piola-Kirchhoff stress tensor) is expressed as

(5) $$\sigma_{x}\left(t\right)=\frac{\partial \Psi }{\partial \varepsilon_{x}}=\int_{0}^{t}G\left(t-\tau \right)\frac{\partial \sigma_{x}^{\left(e\right)}}{\partial \varepsilon_{x}}{\dot{\varepsilon }}_{x}\left(\tau \right)\;d\tau$$

## 3. Results

**Figure 2:**
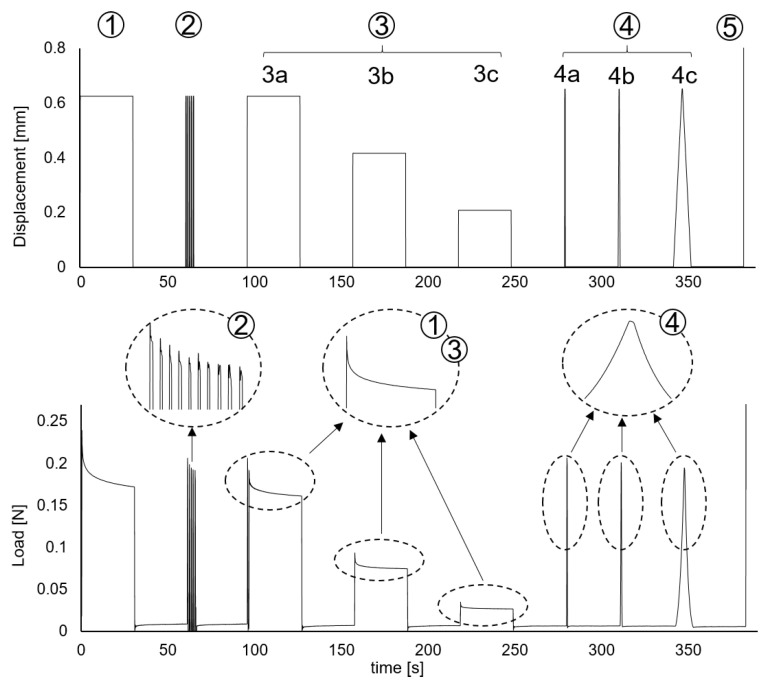
Imposed displacements in the different test stages and reaction force of the PSBV for specimen 2632A/20. The stages with constant displacement have been enlarged to show the decrease in force in stages (1) to (3), and the concavity of the load–discharge curve of stage (4).

For this study, cyclic and uniaxial relaxation tests were performed on six PSBV specimens. Figure 2 clarifies the five stages of the testing process for each specimen. The same figure shows the variation of the imposed displacements over time, together with the axial force measured by the load cell.

Stages (1) and (3) are stages with constant strain: the first one up to the established strain level $\varepsilon_{\max}$, and the second one divided in three stages up to $\varepsilon_{\max}$, $2/3\cdot \varepsilon_{\max}$ and $1/3\cdot \varepsilon_{\max}$. In both stages, while keeping constant the applied displacement and, therefore, the strain, a clear relaxation of the reaction force of the PSBV specimen is observed, with a drop of exponential type, which is a clear indication of the presence of viscoelastic effects in the mechanical behavior of the specimen.

Likewise, stage (2) in which successive loading and unloading cycles have been applied up to $\varepsilon_{\max}$ at high speed, the same behavior is perceived, showing a progressive drop in the force as the number of accumulated cycles increases.

Finally, stage (4) is divided into three load-unload stages at speeds of 0.01,0.1 and 1 s${}^{-1}$ respectively. It can be seen that, the loads being triangular and applied at a constant velocity, the force response is clearly a concave curve, which also indicates the presence of viscoelasticity. Thus, the results obtained from the cycling and relaxation tests indicate that the bridging vein is a viscoelastic material; therefore, a constitutive model that contemplates such viscoelasticity is required to describe its mechanical behavior.

### 3.1. Relaxation Tests

The parameters of the viscoelastic model of Equation (5) were fitted from the data of stages (1) and (3) of Figure 2. For this purpose, the set of force-time values from the instant at which the maximum displacement is reached until the end of the stage was considered.

The axial stress ($\sigma_{x}$) is expressed in terms of the force as $\sigma_{x}\left(t\right)=F_{t}/\lambda_{t}A_{0}$, see Equation (3). Given the low level of deformation, (ε<5%), the material can be considered linear elastic [23] without making large errors and employing the following relationship:

(6) $$\frac{F_{t}}{EA_{0}}=\lambda_{t}\left[\varepsilon_{x}\left(t\right)+\sum_{k=1}^{N}g_{k}\int_{0}^{t}e^{-\left(t-\tau \right)/\tau_{k}}{\dot{\varepsilon }}_{x}\left(\tau \right)\;d\tau \right]$$

Note that, during the initial instants of loading, ${\dot{\varepsilon }}_{x}\neq 0$ and that makes the integral not identically zero (see that once the maximum displacement ${\dot{\varepsilon }}_{x}=0$ is reached, the integral term is null). However, at the end of the stage, the specimen is completely relaxed, so the viscoelastic contribution (VC) ends up being zero. Moreover, from practically the beginning of the trial, both $\lambda_{t}$ and $\varepsilon_{x}$ are constants: $\lambda_{t}=\lambda_{\infty }$ and $\varepsilon_{x}\left(t\right)=\varepsilon_{\max}$. All this implies that the quotient $F_{t}/EA_{0}$ also tends to the constant given by $\lambda_{\infty }\varepsilon_{\max}$. Thus, measuring the force $F_{\infty }$ at the end of the stage provides a way to determine the value of $EA_{0}$ from the ratio $EA_{0}=F_{\infty }/\left(\lambda_{\infty }\varepsilon_{\max}\right)$. For that reason, calculations can be made without a direct measurement of the elastic modulus or cross-section of the specimen by simply using the normalization $f_{t}=F_{t}/EA_{0}$, where the latter magnitude $f_{t}$ will be called reduced force.

**Figure 3:**
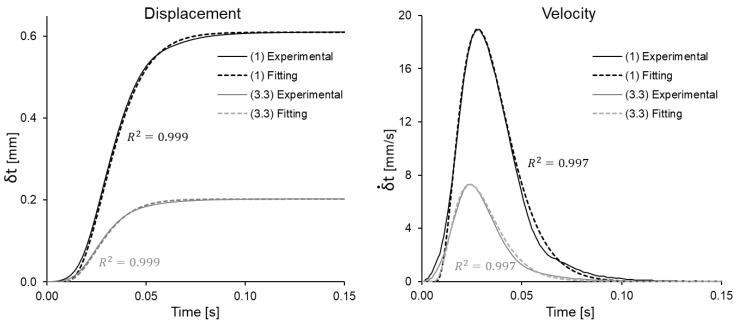
Experimental displacement and experimental velocity applied to a specimen, plotted together with the fit by gamma-type function. The plot also shows the derivatives in Section 1 and Section 3. It can be seen that the fit is in both cases more extreme than 0.997.

During the relaxation itself, $\lambda_{t}$ and, therefore, also $\varepsilon_{x}$ are constant. However, before reaching their constant value, there is a very fast sudden stretching, during which the elongation is given by $\lambda_{t}=1+\delta_{t}/\ell_{0}$, with $\delta_{t}$ being the displacement and the stretching rate being ${\dot{\lambda }}_{t}={\dot{\delta }}_{t}/\ell_{0}$. Experimentally, it was observed that the fast stretching velocity process can be adequately described by a function of “gamma distribution” type [24], with the form

(7) $${\dot{\delta }}_{t}=\frac{\Delta_{\max}}{t_{a}}{\left(\frac{t}{t_{a}}\right)}^{n}\frac{e^{-t/t_{a}}}{\Gamma (n+1)}$$

where $t_{a}$ is a parameter related to the time required to reach the maximum displacement and, in practice, is obtained by fitting the measured displacement velocity data. Similarly, several preliminary tests showed that taking n=3 is sufficient to adequately represent the velocity at the beginning of each relaxation stage. Therefore, the displacement $\delta_{t}$ can be obtained by integrating the above expression (see Figure 3).

From the explicit form (7) of the velocity ${\dot{\delta }}_{t}$, we obtain the strain rate as ${\dot{\varepsilon }}_{x}\left(t\right)=\delta_{t}$ which, introduced in Equation (6), leads to the formula:

(8) $$f_{t}=\frac{F_{t}}{EA_{0}}=\lambda_{t}\left[\varepsilon_{\max}+\sum_{k=1}^{N}g_{k}\frac{e^{-\alpha_{1}t/\theta_{k}}}{{\left(1-\theta_{k}\right)}^{4}{\left(1-2\theta_{k}\right)}^{7}}\sum_{m=1}^{11}\theta_{k}^{m}\left(\mu_{m}+\nu_{m}e^{-\alpha_{2}/\theta_{k}}\right)\right]$$

In this formula, $\alpha_{1}$ and $\alpha_{2}$ are real numbers that depend on the initial conditions of the specimen (length) and the test velocity curve (time of load application, etc.). In the same way, the explicit analytical integration yields the real numbers $\mu_{m}$ and $\nu_{m}$ (the same calculation shows that $\nu_{m}>0,\mu_{m}<0$ for even *m* and $\nu_{m}<0,\mu_{m}>0$ if *m* is odd, and furthermore one has, $\mu_{1},\mu_{2},\mu_{3},\mu_{4}=0$). The only adjustable parameters in the above expression are $\theta_{k}=\tau_{k}/t_{a}$ and $g_{k}$ which are dimensionless. The relaxation times are obtained as $\tau_{k}=\theta_{k}t_{a}$. The number of adjustable parameters is 2N, where *N* is the number of terms in the Prony series, see Equation (4).

**Figure 4:**
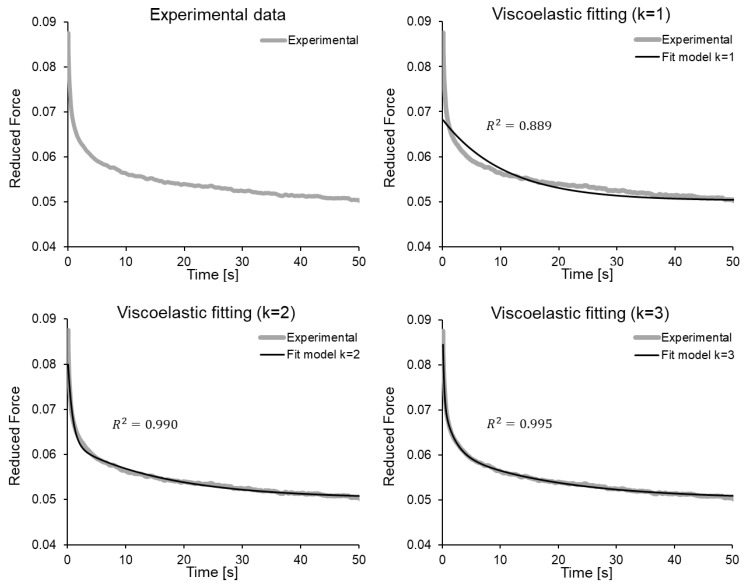
Fitting of the proposed viscoelastic model, for stage (1) of one of the specimens. Fits with N=1, N=2 and N=3 terms in the Prony series are considered, with the corresponding parameters from Table 1.

Figure 4 shows the measured force data for stage 1 of one of the specimens, and the corresponding fits with the viscoelastic model for N=1, N=2 and N=3 in the Prony series (4). The fitted parameters have been set out in Table 1. It can be seen that as *N* increases, the fit improves markedly as expected, since the number of adjustable $g_{k}$ and $g_{k}$ parameters increases:

1. Notice that, although for N=1 the coefficient $r^{2}=0.889$ is quantitatively adequate, qualitatively it is observed that the fit does not adequately represent the vertical asymptote of force drop, nor the curve in general.
2. With N=2 there is a marked improvement in the fit ($r^{2}=0.990$).
3. Finally, for N=3 ($r^{2}=0.995$), both the asymptote and the curve are adequately represented by the viscoelastic model.

Thus, in this study the parameters $g_{k}$ and $\tau_{k}$ have been determined for N=3$\left(g_{1},g_{2},g_{3};\tau_{1},\tau_{2},\tau_{3}\right)$. The viscoelastic model fits have been repeated for stages (1) and (3), the latter divided into subplots (3.1), (3.2) and (3.3), thus obtaining the parameters. The average for each specimen is presented in Table 2.

It can be observed in the table that the higher the value of $g_{k}$ (from $g_{1}$ to $g_{3}$), the lower the value of $\tau_{k}$, thus increasing the degree of contribution of the model term, and the shorter the relaxation time. The relaxation times decrease by an order of magnitude progressively between $\tau_{1}$ and $\tau_{3}$, obtaining similar values among the different specimens. The average characteristic times for the sample are $\tau_{1}=17.22\pm 5.17$ s, $\tau_{2}=1.49\pm 0.55$ s and $\tau_{3}=0.13\pm 0.05$ s. The average values of the contribution coefficients for the sample are $g_{1}=0.17\pm 0.06$, $g_{2}=0.17\pm 0.08$ and $g_{3}=0.87\pm 0.62$.

**Figure 5:**
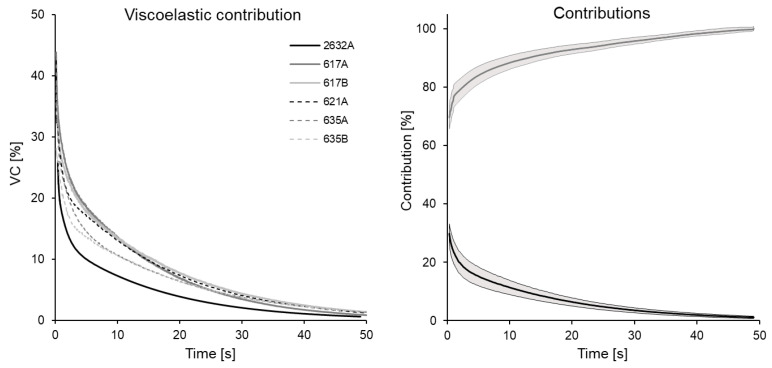
Evolution of VC in strength over time for the analyzed specimens: the black lines indicate the average elastic and viscoelastic contributions as a function of time, the shaded area indicates the error interval in the sample.

In addition, to show the importance of the viscoelastic effect in PSBVs, the VC for the reduced force has been calculated, i.e., what percentage of the response corresponds to the elastic effect and what amount to the viscoelastic one. The VC for all specimens, calculated from the parameters $g_{k}$ and $\tau_{k}$ in Table 2, is shown in Figure 5. It can be seen that at the initial instant, the VC is between 26 and 35%, decreasing exponentially until it reaches approximately 0% at the end of the relaxation stretch. In fact, after 15 s of relaxation, the specimen has relaxed to a great extent and the VC is less than 10% in all samples.

Likewise, the average VC and viscoelasticity and the confidence interval have been determined from the parameters of the studied specimens (Figure 5). Thus, it can be seen that initially the viscoelastic part corresponds to 29.8 ± 3.35%, decreasing to 11.4 ± 2.40% at 10 s and 4.8 ± 1.06% at 25 s, while the elastic contribution starts from 69.8 ± 4.03%, rising to 88.5 ± 2.64% at 10 s to 94.9 ± 1.10% at 25 s.

### 3.2. Fast Loading/Unloading Cycle Tests

The viscoelastic effect can also be analyzed in test stage (2), in which fast loading and unloading cycles have been performed. These cycles are fast compared to some of the relaxation times $\tau_{k}$ of the material. This leads to the assumption that in the time in which a loading and unloading cycle elapses, the BV may not have enough time to relax the internal stresses and this is reflected in the maximum forces reached in each cycle.

**Figure 6:**
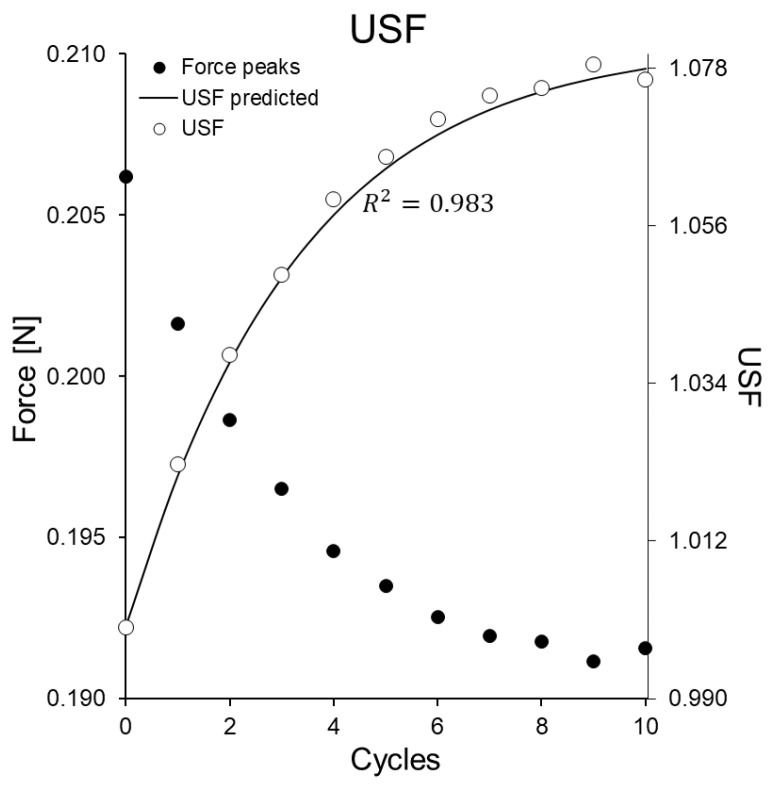
Force peaks in stage (2) as a function of the number of cycles performed and USF computed and predicted by Equation (10).

Figure 6 shows the force peaks in successive cycles. It can be seen how in each new cycle the maximum force reached is lower, showing a progressive relaxation of the tissue of the PSBV as the number of cycles increases. For each sample, the USF (unconditioned scale factor), defined in [12], has been calculated as the following ratio:

(9) $$\mathrm{USF}\left(n,T\right)=\frac{F_{1}}{F_{n}}>1$$

where *n* is the cumulative cycle number and *T* is the period of the cycles (T=0.55). As can be seen in the figure above, the magnitude of the USF grows progressively with the number of cycles. In some samples, it has reached 20 cycles, while in others it has been limited to 10 cycles. From the results, it has been calculated that the USF(n=10)=1.14±0.085 and USF(n=20)=1.23±0.095. In Appendix A, it is justified that the USF should be able to be approximated by a function of the type

(10) $$\mathrm{USF}\left(n,T\right)\approx \frac{1+g_{0}}{1+g_{0}e^{-\alpha (T)n}}$$

This equation has the form of Equation (A7) and that prediction is just what is observed in Figure 6, where the fit between the above formula and the experimental data is very good ($r^{2}=0.983$). The results obtained for the parameters in the sample set are $g_{0}=0.206\pm 0.047$ and α=0.127±0.028.

### 3.3. Load-Unload Tests

**Figure 7:**
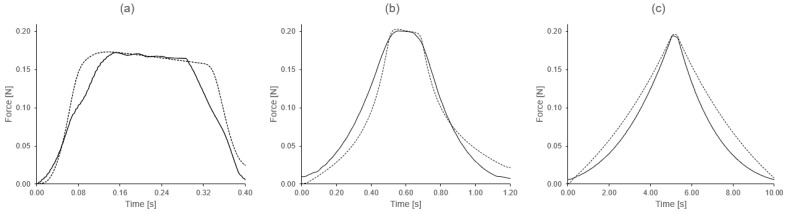
Experimental force–time curves compared with model predictions: (**a**) fast curve ($\dot{\varepsilon }\approx 1$ s${}^{-1}$), (**b**) medium velocity curve ($\dot{\varepsilon }\approx 0.1$ s${}^{-1}$) and (**c**) slow curve ($\dot{\varepsilon }\approx 0.01\;$ s${}^{-1}$). The predicted curve has been obtained by numerical integration. The cumulative integration error is larger for the slow curve and smaller for the fast curve. However, the fast curve seems to have a larger measurement error. Possibly, for that reason, the best fit is obtained for the medium speed curve.

In addition to relaxation tests and fast loading cycle tests, three additional loading and unloading tests were performed in the form of triangular waves at very different strain rates, corresponding to stages 4a, 4b and 4c performed at constant stretching speeds ${\dot{\lambda }}_{t}=0.01,0.1$ and 1 s${}^{-1}$. The main purpose was to verify whether the characteristic times ($\tau_{k}$) and contribution coefficients ($g_{k}$) obtained from the relaxation tests could well explain the shape of these curves. The displacement had a very roughly triangular shape (except for brief periods of acceleration and deceleration), each triangular wave included about 350 measurements along the loading and unloading cycle ($N_{p}$ = 285, 365 and 385 points, respectively), that allowed making a numerical integration of the viscoelastic contribution in (6), using Simpson’s 3/8 rule [25]:

(11) $$\begin{array}{cc} I_{k} & :=\int_{0}^{t=N_{p}h}\overset{f(\tau )}{\overbrace{e^{\tau /\tau_{k}}{\dot{\varepsilon }}_{x}\left(\tau \right)}}\;d\tau \approx \\ \; & =\frac{1}{4}\sum_{k=0}^{N_{p}-3}\frac{3h}{8}\left(f\left(t_{k}\right)+3f\left(t_{k+1}\right)+3f\left(t_{k+2}\right)+f\left(t_{k+3}\right)\right) \end{array}$$

where $h=t/N_{p}$ and $t_{k}=kh$. The result can be seen in Figure 7 where the measured and model-predicted force curves are shown. The fit is reasonable although the medium and low velocity curves show larger divergences from the model.

This could be partly due to the fact that the integration error is larger for the slower loading curves. This happens because the number of points is similar in the three curves, but the duration $T_{w}$ of the triangular wave is different, the numerical error depends on the time step *h* used for the numerical integration, which is given by the relation $h=T_{w}/N_{p}$ ($N_{p}$ being similar for the three curves). The error resulting from Simpson’s 3/8 rule of integration is given by:

(12) $$e\leq \frac{3h^{5}}{80}N_{p}\sum_{k}\max_{\xi_{k}\in \left(t_{k-1},t_{k}\right)}\frac{d^{4}f\left(\xi_{k}\right)}{d\tau^{4}}<C\;\frac{3T_{w}^{5}}{80N_{p}^{4}}\dot{\varepsilon }$$

So, for the faster curve a smaller error was made, while for the slower curve the integration error was larger (having a similar number of points, but the duration of the last wave being longer, which makes the division coarser $h=T/N_{p}$).

## 4. Discussion

The mechanical behavior of PSBVs has been previously studied by some researchers [5,7,9,26], although most of the previous studies do not explicitly consider viscoelastic effects in the stress/force–strain curves [10]. Some strain rate related effects are reported, especially with respect to the influence of strain rate on the mechanical failure of PSBVs [3,4,6,7]. Interestingly, like other biological tissues, PSBVs appear to exhibit failure stresses negatively correlated with strain rate [10,27].

However, the relaxation and fast-cycle tests performed in this study clearly show a viscoelastic effect in the PSBV response, especially in stress relaxation stages under constant strain, where the force on the PSBVs is progressively reduced. This fact had not been investigated in detail in the literature on mechanical properties of PSBV [9], although viscoelastic effects had been measured in rapid load cycles [10].

Regarding the results obtained, this work is possibly the first study of PSBVs estimating the necessary relaxation times of the Prony series, see Equation (4), for modeling the viscoelastic behavior of PSBVs. Interestingly, excellent fits ($r^{2}>0.99$) are obtained with three relaxation times and these differ by an order of magnitude, as $\tau_{1}=O\left(10^{1}\right),\tau_{2}=O\left(10^{0}\right),\tau_{3}=O\left(10^{-1}\right)$, so each of them seems to capture a different time scale. This is in agreement with the findings of Funk et al. (2000) [12], where the viscoelasticity of ankle ligaments is studied and where the observed scales are $\tau_{1}=O\left(10^{2}\right),\tau_{2}=O\left(10^{1}\right),\tau_{3}=O\left(10^{0}\right)$. On the other hand, in Davis & De Vita (2012) [13], it is pointed out that the QLVE model applied to rat tendons only captures the behavior well for t<10 s, which is why they propose a nonlinear viscoelastic constitutive model that overcomes the shortcomings of QLVE to explain their data.

As for the analysis of the preconditioning, which is based on the USF given by Equation (9), an analysis based on viscoelastic behavior has been presented here, which allows predicting that this magnitude will vary from one cycle to another by means of Equation (10). With only two parameters, this formula predicts a better fit than the exponential type heuristic formula:

(13) $${\mathrm{USF}}_{n}=a_{0}+\sum_{k=1}^{3}a_{k}e^{-b_{k}n}$$

used in [10] and previously in [28,29,30]. Although the Formula (13) is numerically adequate, it is a heuristic formula and is not directly derived from the viscoelastic behavior equations unlike the Formula (10).

As for the applications of this work, they can be grouped into three different areas:

1. Clinicians are charged with the significant task of distinguishing between accidental and inflicted head trauma. Some times this distinction is straightforward, but in many cases the probabilities of injuries from accidental scenarios are unknown, making the differential diagnosis difficult [31]. A refinement of the knowledge of the tolerance ranges against rupture of PSBVs may provide greater accuracy in the reconstruction of injury mechanisms.
2. Computational biomechanics can simulate many potentially traumatic situations, so that models already allow detailed reconstructions of the sequence of events leading to a severe SDH. Accurate knowledge of the material behavior can improve FEHMs, as their inaccuracy is often not so much a computational problem, but a poor characterization of the biomechanical behavior of brain structures. For example, a good number of FEHMs use a stress-strain response for PSBVs that does not reflect the measured nonlinear behavior [3], e.g., the UDS FEHM (Université de Strasbourg) [32], the KTH FEHM (KTH Royal Institute of Technology) [33], UCDBTM (University College Dublin) [34,35], WSUBIM (Wayne State University) [36] or G/LHM [37] also model PSBVs as elastic beams with a linear stress-strain response [9]. The recognition of the importance of the nonlinear behavior and viscoelasticity of brain structures has been explicitly pointed out by the developers of YEAHM (University of Aveiro) [38] and interestingly, some FEHMs model PSBVs as nonlinear elastic materials [39]. In particular, Equation (4), together with the averages obtained from Table 2, allow us to compute an estimation of the viscoelastic effect, independent of the starting elastic model for the PSBVs, which is being used in the FEHM.
3. The improvement of injury metrics used to assess restraint systems in vehicles or the design of other preventive elements against head trauma. Currently, the estimation is often done by the injury metric called “relative motion damage measure” (RMDM) [40,41], used to predict the probability of a SDH due to the failure of PSBVs [42,43]. However, that metric was developed based on obsolete data [26], and the data from this study can be used to update that injury metric.

Finally, some current limitations of the present study that could be improved in future work:

1. The sample used is consistent but small. So, the effects of age, gender, or other anthropometric characteristics on the viscoelastic mechanical properties could not be determined.
2. In addition, a QLVE model has been used in which the relaxation function is separable, in the sense of [13]. Given the low strain levels used for the tests (since care was taken not to cause irreversible damage to the specimen from one test stage to the next), no effects of non-separability were found. However, further work could build a somewhat more general model on that basis. In any case, the proposed model is a first approximation that even explains the data that were not used for the fits (see Section 3.3).
3. Moreover, a further extension of this work would be to examine whether the relaxation curves could be modeled, using the Prony series of stretched exponential relaxation [44]. This could lead to series with fewer and/or more accurate terms, although it is not clear if this is the case. Further work is needed in order to determine whether the use of stretched exponentials or a more general non-separable viscoelastic model would provide better models.

## 5. Conclusions

The results reported in the current study are the first complete characterization of the viscoelastic response of PSBVs. Relaxation times and coefficients of the Prony series are computed allowing the use of these values in combination with any hyper-elastic model for PSBVs. The model explains accurately all the load cases considered.

In addition, the fitted constitutive parameters can be used for computational FEHMs; these models and the obtained values could be used to reconstruct the circumstances of head trauma and for assessing the causes of TBI. Finally, the results of this study are highly relevant to assess acute SDH due to the mechanical failure of PSBVs.

## Figures and Tables

**Table 1:** Parameters $g_{k}$, $\tau_{k}$ of the viscoelastic model obtained for the specimen 635/21B for different values of *k*.

*N*	$g_{1}$ [–]	$g_{2}$ [–]	$g_{3}$ [–]	$\tau_{1}$ [s]	$\tau_{2}$ [s]	$\tau_{3}$ [s]
N=1	0.360	—	—	10.756	—	—
N=2	0.243	0.393	—	13.397	0.799	—
N=3	0.215	0.221	0.398	18.162	1.885	0.211

**Table 2:** Average ± standard deviation of the parameters $g_{1},g_{2},g_{3}$ and $\tau_{1},\tau_{2},\tau_{3}$ of the viscoelastic model in the four relaxation stages obtained for each specimen.

Specimen	$g_{1}$ [–]	$g_{2}$ [–]	$g_{3}$ [–]	$\tau_{1}$ [s]	$\tau_{2}$ [s]	$\tau_{3}$ [s]
2632A	0.10 ± 0.05	0.13 ± 0.04	1.70 ± 0.29	10.96 ± 2.64	0.88 ± 0.19	0.07 ± 0.01
617A	0.21 ± 0.10	0.32 ± 0.16	0.34 ± 0.06	15.38 ± 4.90	1.99 ± 2.62	0.17 ± 0.19
617B	0.24 ± 0.07	0.13 ± 0.08	0.30 ± 0.23	13.78 ± 9.15	0.96 ± 0.70	0.09 ± 0.08
621A	0.20 ± 0.07	0.18 ± 0.10	0.48 ± 0.42	17.08 ± 16.11	1.20 ± 1.23	0.09 ± 0.10
635A	0.12 ± 0.07	0.13 ± 0.07	0.84 ± 1.13	20.73 ± 13.34	2.16 ± 1.46	0.20 ± 0.10
635B	0.14 ± 0.06	0.16 ± 0.08	1.60 ± 1.62	25.41 ± 8.22	1.76 ± 1.93	0.17 ± 0.20

## Data Availability

Due to the restrictions imposed by the legal collaboration agreement between IMLFC and UPC, the data are not publicly available, although they can be accessed upon non-anonymous request to the authors of the study and under the conditions set by the aforementioned legal collaboration agreement.

## Abbreviations

The following abbreviations are used in this manuscript:

PSBV(s)	Parasagittal Bridging Vein(s)
FEHM	Finite Element Head Model
QLVE	Quasi-Linear Viscoelastic
SDH	Subdural Hematoma
TBI	Traumatic Brain Injury
VC	Viscoelastic Contribution

## Appendix A. Prediction of the USF

In this appendix. we deduce what behavior is to be expected from USF(n,T), defined in the Equation (9), and we justify mathematically why we expect a behavior given by Formula (10). The displacement $\delta_{t}$ in the cyclic test is periodic, therefore, it can be expressed by a trigonometric Fourier series:

(A1) $$\delta_{t}=\Delta \delta \left[\frac{a_{0}}{2}+\sum_{m=1}^{\infty }a_{m}\cos\left(\frac{2m\pi }{T}t\right)+\sum_{m=1}^{\infty }b_{m}\sin\left(\frac{2m\pi }{T}t\right)\right]$$

where T=0.55 s is the period of the cyclic stretch. The stretch $\lambda_{t}$, its corresponding rate ${\dot{\lambda }}_{t}$, the strain $\varepsilon_{x}$ and the strain rate ${\dot{\varepsilon }}_{x}$ can be calculated with the following formulas:

(A2) $$\lambda_{t}=1+\frac{\delta_{t}}{\ell_{0}},\;{\dot{\lambda }}_{t}=\frac{{\dot{\delta }}_{t}}{\ell_{0}},\;\varepsilon_{x}=\frac{\lambda_{t}^{2}-1}{2},\;{\dot{\varepsilon }}_{x}=\lambda_{t}{\dot{\lambda }}_{t}$$

To estimate the viscoelastic effect we consider a finite sequence of integrals of the form

(A3) $$J_{n}^{\left(k\right)}:=\int_{0}^{t_{n}}e^{-\frac{t_{n}-\tau }{\tau_{k}}}\frac{{\dot{\delta }}_{\tau }}{\ell_{0}}\left(1+\frac{\delta_{\tau }}{\ell_{0}}\right)\;d\tau$$

where $t_{n}=\left(n-1+\gamma \right)T$ are the times for which successive force peaks occur:

(A4) $$J_{n}^{\left(k\right)}\left(T\right)=\sum_{m=1}^{\infty }\frac{a_{m}P_{1,m}\left(\theta_{k}\right)+b_{m}P_{2,m}\left(\theta_{k}\right)+\left[a_{m}P_{3,m}\left(\theta_{k}\right)+b_{m}P_{4,m}\right]e^{-\left(n-1+\gamma \right)\frac{T}{\tau_{k}}}}{\left(1+4m^{2}\theta_{k}^{2}\right)\left(1+16m^{2}\theta_{k}^{2}\right)}$$

where $\theta_{k}=\pi T/\tau_{k}$ has been introduced for brevity, and further we have $P_{j,m}\left(\theta_{k}\right)$ as easily computable fourth-degree polynomials, whose coefficients depend on cos(mγπ) and sin(mγπ). The above formula allows us to compute successive force peaks and, thus,

(A5) $$\mathrm{USF}\left(n,T\right)=\frac{1+\sum_{k=1}^{N}g_{k}J_{1}^{\left(k\right)}\left(T\right)}{1+\sum_{k=1}^{N}g_{k}J_{n}^{\left(k\right)}\left(T\right)}$$

where the $g_{k}$ are the same coefficients that appear in the Equation (6) and *j* is the index for which $\left|T-1,5\tau_{j}\right|$ is minimum. It can be seen that the value of USF approaches a maximum when the $\theta_{j}\in \left(4,5\right)$ (the exact position of the maximum depends on the $g_{k}$, although very little). Taking the Equation (A4) into consideration, the expression USF(n,T) would have the form

(A6) $$\mathrm{USF}\left(n,T\right)=\frac{1+\sum_{k=1}^{N}g_{k}\left[c\left(\theta_{k}\right)+d\left(\theta_{k}\right)\right]}{1+\sum_{k=1}^{N}g_{k}\left[c\left(\theta_{k}\right)+d\left(\theta_{k}\right)e^{-n\frac{T}{\tau_{k}}}\right]}=\frac{1+\sum_{k=1}^{N}{\tilde{g}}_{k}}{1+\sum_{k=1}^{N}{\tilde{g}}_{k}e^{-n\frac{T}{\tau_{k}}}}$$

where $c(\theta_{k})$ and $d(\theta_{k})$ are functions which can be computed from Equation (A4), and where the ${\tilde{g}}_{k}$ are given by

$${\tilde{g}}_{k}=\frac{g_{k}d\left(\theta_{k}\right)}{1+\sum_{k=1}^{N}g_{k}c\left(\theta_{k}\right)}$$

The expression (A6) is exact, but inconvenient, in fact, numerically it can be seen that the summand for which it is satisfied that $\left|T-1.5\tau_{j}\right|$ is minimal, so a reasonable approximation can be obtained using only one characteristic time:

(A7) $$\mathrm{USF}\left(n,T\right)\approx \frac{1+g_{j}J_{1}^{\left(j\right)}\left(T\right)}{1+g_{j}J_{n}^{\left(j\right)}\left(T\right)}=\frac{1+{\tilde{g}}_{j}}{1+{\tilde{g}}_{j}\exp\left(-n\frac{T}{\tau_{j}}\right)}$$
